# A Case of Hernia

**Published:** 1789

**Authors:** Thomas Clowes

**Affiliations:** Member of the Corporation of Surgeons of London, and Surgeon at Wingham, in Kent


III. *A Caſe of Hernia. Communicated in a Letter to Dr.* Simmons, *F. R. S. by Mr.* Thomas Clowes, *Member of the Corporation of Surgeons of London, and Surgeon at Wingham, in Kent.*

ON Wedneſday, the 23d of July laſt, I was deſired to viſit Robert Gifford, a labouring man, ſixty-three years old, at Ickham, a village near this place. Upon inquiring into his complaints, I found he had, for ſeveral years, been troubled with an inguinal rupture on the right ſide, but which had never deſcended lower than the groin, and, till now, had always been reduced, without difficulty, when he lay upon a bed.

The

The tumour in the groin was of about the fize of an hen's egg, and, from the violence he had ufed in his endeavours to reduce it, was become fo tender, that he could fcarcely fuffer me to touch it. As he had been feveral hours in this ftate, and without any evacuation by ftool, I directed (after bleeding him) a purgative mixture and a clyfter to be adminiftered. At the end of fix hours, when I vifited him again, I found him much relieved. He had had feveral ftools, and the tumour was fomewhat diminifhed.

On Saturday the 26th, being informed that he was again coftive, I ordered the purgative medicine to be repeated, and the day following, when I vifited him, found it had given him as much relief as before; but as the tumour was ftill irreducible, and he complained of pain when he walked, I fuppofed an adhefion might be taking place.

I faw him twice in the following week as I paffed by his houfe, and found him each time walking about, and complaining of pain only when he ufed too much exercife. In this ftate, however, he did not long continue; for on Tuefday the 5th of Auguft I was called to him in great hafte, and was informed that he had been in violent pain during the whole of the preceding night. On examining the tumour in the groin, I found the integuments were on the point of breaking, which they did before the next morning. The opening was about the ſize of a crown piece; the hernial ſac and omentum protruded at the wound; the inteſtine was ruptured, and the fæces were diſcharged at the opening. On removing the omentum, I diſcovered that the opening in the gut was longitudinal, and at its anterior part only.

Almoſt the whole of the fæces continued to be diſcharged at the wound for a fortnight, ſo that I feared leſt the inteſtine ſhould become ſo much diſeaſed as to be totally divided; in which caſe this part muſt, of courſe, have remained as the anus ever after. I therefore deſired a neighbouring Surgeon* of eminence might be conſulted. He approved of the plan of treatment I was purſuing, and agreed with me in opinion that no operation could have been undertaken with propriety in any ſtage of the diſeaſe.

Clyſters were adminiſtered daily, and ſometimes a ſmall quantity of fæces came way with

* Mr. Loftie, of Canterbury.

them.

them. The wound was dreſſed as often as circumſtances required, and the parts were well fomented. The patient thought that, at times, wind paſſed by the anus, as it did frequently at the wound.

About the 20th of Auguſt the fæces began to take their natural courſe more freely, and in another week from that time the healing of the wound was ſo far completed as to prevent any farther diſcharge that way. At the beginning of September he went out to pick hops, and followed that employment upwards of a month without any inconvenience. The wound has now been entirely healed ſeveral weeks, and he follows his former occupation of thraſhing, and is as well as before his late illneſs. During the cure he took a few ounces of Peruvian bark in decoction.

This caſe, ſo far as relates to a ſmall part of the inteſtine being engaged, reſembles one related by the late ingenious Mr. Elſe in the fourth volume of the Medical Obſervations and Inquiries. In that caſe a fatal ſtrangulation enſued, though the whole circumference of the gut was not encloſed in the ſtricture; and in my patient, if the whole of the inteſtine had been engaged, either a ſimilar event or a total

ſeparation of the gut muſt have been the conſequence.

*Wingham,*

December 7, 1788.

